# The Role of Macrophage Populations in Skeletal Muscle Insulin Sensitivity: Current Understanding and Implications

**DOI:** 10.3390/ijms241411467

**Published:** 2023-07-14

**Authors:** Min-Kyeong Lee, Heeyeon Ryu, Ji Yun Van, Myeong-Jin Kim, Hyeon Hak Jeong, Won-Kyo Jung, Joo Yun Jun, Bonggi Lee

**Affiliations:** 1 Department of Food Science and Nutrition, Pukyong National University, Busan 48513, Republic of Korea; 3633234@hanmail.net (M.-K.L.); heeyeon3115@naver.com (H.R.);; 2 Department of Smart Green Technology Engineering, Pukyong National University, Busan 48513, Republic of Korea; bandallym@gmail.com (J.Y.V.);; 3 Division of Biomedical Engineering and Research Center for Marine Integrated Bionics Technology, Pukyong National University, Busan 48513, Republic of Korea; wkjung@pknu.ac.kr; 4 Neuroscience and Cognitive Science Program, University of Maryland, College Park, MD 20742, USA; jyjun@gmail.com

**Keywords:** macrophage populations, skeletal muscle, type 2 diabetes (T2D), insulin sensitivity

## Abstract

Insulin resistance is a crucial factor in the development of type 2 diabetes mellitus (T2DM) and other metabolic disorders. Skeletal muscle, the body’s largest insulin-responsive tissue, plays a significant role in the pathogenesis of T2DM due to defects in insulin signaling. Recently, there has been growing evidence that macrophages, immune cells essential for tissue homeostasis and injury response, also contribute to the development of skeletal muscle insulin resistance. This review aims to summarize the current understanding of the role of macrophages in skeletal muscle insulin resistance. Firstly, it provides an overview of the different macrophage populations present in skeletal muscle and their specific functions in the development of insulin resistance. Secondly, it examines the underlying mechanisms by which macrophages promote or alleviate insulin resistance in skeletal muscle, including inflammation, oxidative stress, and altered metabolism. Lastly, the review discusses potential therapeutic strategies targeting macrophages to improve skeletal muscle insulin sensitivity and metabolic health.

## 1. Introduction

Type 2 diabetes (T2D) is a chronic condition in which the body is unable to properly use and store glucose, leading to high levels of glucose in the bloodstream. It is the most common form of diabetes and is typically diagnosed in adults, although it can occur in children as well [1]. In T2D, the body becomes resistant to insulin, a hormone that regulates the amount of glucose in the blood. As a result, the pancreas may produce more insulin to compensate, but eventually, it may not be able to keep up with the demand. This leads to high levels of glucose in the bloodstream, which can cause a range of health problems over time [1].

Skeletal muscle plays a critical role in glucose homeostasis, as it is responsible for the majority of glucose uptake in response to insulin. However, insulin resistance in skeletal muscle is a hallmark of T2D, and the mechanisms underlying this resistance are complex and multifactorial. In recent years, there has been growing interest in the role of macrophages in the development of insulin resistance in skeletal muscle [2]. Macrophages are immune cells that can infiltrate various tissues in response to inflammation and contribute to the pathogenesis of metabolic disorders. In skeletal muscle, different populations of macrophages have been identified, each with their unique functions and roles in insulin sensitivity [3]. Understanding the interplay between macrophages and skeletal muscle is crucial for developing novel therapeutic strategies to combat insulin resistance and related metabolic disorders. In this review, we will provide an overview of the different macrophage populations present in skeletal muscle and their functions, discuss how macrophages infiltrate skeletal muscle during obesity and contribute to insulin resistance, describe the underlying mechanisms by which macrophages promote or ameliorate insulin resistance in skeletal muscle, and highlight future directions for research in this area. Ultimately, we aim to provide insights into the potential of targeting macrophages as a therapeutic strategy for the treatment of T2D and related metabolic disorders.

## 2. Skeletal Muscle and Glucose Homeostasis

Skeletal muscle is the largest organ system in the body, accounting for about 40% of total body weight in young adults [4]. It plays a vital role in movement, posture, thermogenesis, glucose homeostasis, soft tissue support, and metabolism. Skeletal muscle is composed of bundles of muscle fibers, which can be classified into different types based on their metabolic characteristics and expression of myosin heavy chain (MHC) isoforms [2,5]. There are two main types of skeletal muscle fibers: slow-twitch (Type I) and fast-twitch (Type II). Slow-twitch fibers are highly oxidative and rely primarily on aerobic metabolism to generate energy, which makes them well-suited for endurance activities. Fast-twitch fibers, on the other hand, are more glycolytic and rely mainly on anaerobic metabolism to generate energy, which makes them better suited for activities that require short bursts of intense effort [5].

Insulin resistance is a key feature of T2D, and skeletal muscle is the primary site of insulin-stimulated glucose uptake. It is responsible for over 80% of glucose uptake from the bloodstream following a meal, a process that is mediated by insulin [6,7]. Skeletal muscle insulin resistance is characterized by a reduced ability of insulin to stimulate glucose uptake and glycogen synthesis. This leads to decreased glucose disposal and elevated blood glucose levels, which in turn exacerbate insulin resistance and contribute to the progression of T2D [2,6]. Insulin resistance in skeletal muscle is a multifactorial process that involves a combination of genetic and environmental factors, including physical inactivity, excess caloric intake, and inflammation. Especially, chronic low-grade inflammation, also known as meta-inflammation, is now recognized as a key driver of insulin resistance in skeletal muscle. Meta-inflammation can activate various immune cells, including macrophages, which can produce pro-inflammatory cytokines and impair insulin signaling in skeletal muscle [8]. Moreover, macrophages can infiltrate skeletal muscle tissue in response to obesity and insulin resistance, further exacerbating the inflammatory response and impairing glucose uptake [2,8].

At the molecular level, the insulin signaling pathway plays a crucial role in regulating glucose homeostasis in skeletal muscle. When the concentration of glucose in the bloodstream increases, the pancreas releases insulin, which then binds to insulin receptors located on the surface of skeletal muscle cells. This binding event triggers a signaling cascade that ultimately leads to the movement of glucose transporter type 4 (GLUT4) transporters from inside the cell to the plasma membrane, thereby enabling glucose to enter the muscle cells [9,10]. The translocation of GLUT4 transporters is regulated by a complex series of events, including the activation of insulin receptor substrate proteins and the downstream activation of phosphoinositide 3-kinase and protein kinase B (AKT) signaling pathways. These pathways converge to activate a molecular motor called myosin Va, which moves GLUT4-containing vesicles toward the plasma membrane [9,10]. In addition to the insulin signaling pathway, adenosine monophosphate-activated protein kinase (AMPK) is another important regulator of glucose homeostasis in skeletal muscle. AMPK is activated during conditions of energy stress, such as exercise or calorie restriction, and can increase glucose uptake and utilization in skeletal muscle by promoting the translocation of GLUT4 transporters to the plasma membrane [9]. Overall, the insulin–GLUT4 signaling pathway and the AMPK pathway are critical for maintaining glucose homeostasis in skeletal muscle by ensuring that glucose is efficiently taken up and utilized by the muscle cells.

## 3. Macrophage Populations in Skeletal Muscle

### 3.1. Skeletal Muscle-Resident Macrophages

**Figure 1:**
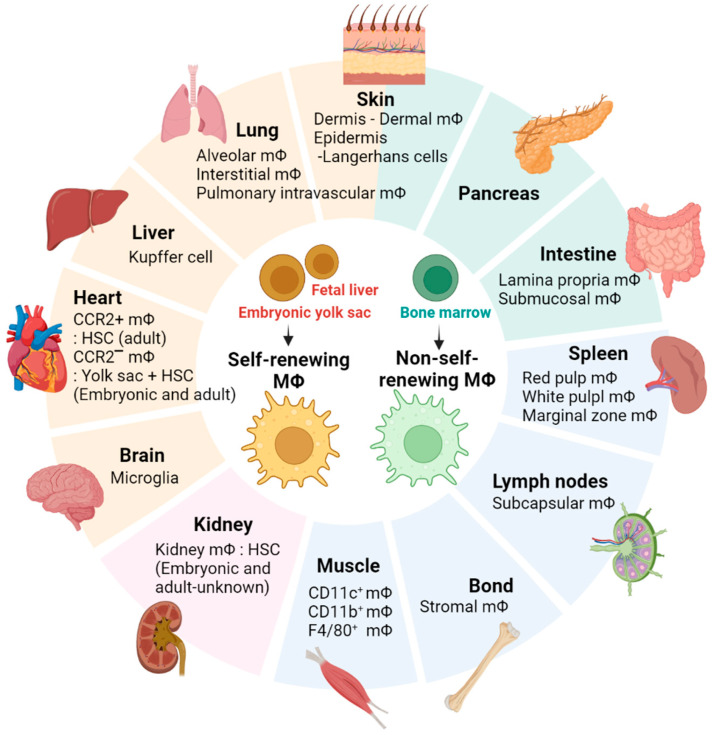
Tissue-resident macrophages. The figure illustrates the distribution of tissue-specific macrophage populations in various body locations.

Macrophages are cells of the innate immune system that serve as the first line of defense against pathogens and are also deeply involved in inflammation, wound healing, dead cell removal, tissue development, maintenance, and remodeling [11,12]. Macrophages can be categorized into two main types: tissue-resident macrophages and -non-resident macrophages [13]. Tissue-resident macrophages can be further classified into two populations: embryo-derived self-renewing macrophages and bone marrow–derived non-self-renewing macrophages [14]. Typical self-renewing macrophages, originating from the embryonic yolk sac or fetal liver, encompass Kupffer cells, alveolar macrophages, microglia, and Langerhans cells (Figure 1) [15,16,17,18]. Non-self-renewing resident macrophages in the skin dermis, intestine, and pancreas are continuously replenished in each tissue by circulating monocytes derived from the bone marrow (Figure 1) [19]. Macrophages are present in virtually every tissue in the body, and local microenvironmental signals in each tissue contribute to the tissue-specific characteristics of resident macrophages [20]. For example, microglia, the brain-resident macrophages, play a crucial role in the elimination of redundant or inappropriate synapses during brain development [21]. Cardiac resident macrophages contribute to the facilitation of electrical conduction in the heart [22], while adipose tissue-resident macrophages are engaged in thermoregulation and the process of lipolysis [23]. Kupffer cells, which are tissue macrophages located in the liver, play a crucial role in liver function by clearing particulate matter from the portal circulation and eliminating or detoxifying microorganisms and toxins that are absorbed from the gastrointestinal tract [24]. Therefore, tissue-resident macrophages are a highly heterogeneous population in terms of phenotype and function, which highlights the need to study macrophage function within the unique microenvironment of the residence tissue.

Macrophages in skeletal muscle are known to play a role in regulating tissue repair and regeneration, but the characteristics of these macrophages have not yet been fully elucidated. Recent studies have provided evidence that the composition of skeletal muscle tissue contains a mixture of self-renewing and non-self-renewing resident macrophages [25]. CD45^+^CD11b^+^F4/80^+^CD64^+^Ly6C^lo^MerTK^+^CD11c^−^CD163^+^CD206^+^ macrophages were detected in skeletal muscle in the steady state and are distributed in interstitial tissues including perimysium, epimysium, and endomysium [25]. These skeletal muscle-resident macrophages are derived from yolk sac hematopoiesis, such as primitive macrophages, fetal liver hematopoiesis, such as fetal monocytes of non-hematopoietic stem cells origin and fetal monocytes of hematopoietic stem cells origin, and postnatal bone marrow hematopoiesis, such as bone marrow hematopoietic stem cells-derived adult monocytes [25]. Compared to the resident macrophage populations of non-muscle tissues, such as peritoneal (CD45^+^F4/80^+^MHCII^−^) [26] and lung alveolar (CD45^+^CD11c^+^Siglec F^+^) macrophages [26], the macrophages residing in the quadriceps (limb muscle) and diaphragm (respiratory muscle) share a common set of differentially expressed genes [25]. A total of 215 genes exhibited significantly higher expression levels in skeletal muscle-resident macrophages than in those residing in non-muscle tissues, with a substantial number of these genes being associated with tissue homeostasis and muscle growth, antigen processing and presentation, as well as chemokine signaling [25]. These findings suggest that macrophages residing in skeletal muscle have the ability to present antigens, support tissue homeostasis, and facilitate muscle growth and regeneration. An analysis focused on transcriptional regulation confirmed that several genes encoding the v-maf musculoaponeurotic fibrosarcoma oncogene homolog, myocyte enhancer factor 2C, and transcription factor 4 were expressed at markedly higher levels in skeletal muscle-resident macrophages than in peritoneal and lung alveolar macrophages [25]. On the other hand, the expression levels of GATA-binding factor 6, a signature transcription factor of peritoneal resident macrophages, and CCAAT/enhancer-binding protein beta, a signature transcription factor of lung alveolar macrophages, were very low in skeletal muscle-resident macrophages [25]. However, whether the v-maf musculoaponeurotic fibrosarcoma oncogene homolog, myocyte enhancer factor 2C, and transcription factor 4 are indeed responsible for tissue-specific transcription in skeletal muscle-resident macrophages requires further studies. Taken together, these results suggest that resident macrophages in different skeletal muscles share functional similarities while being distinct from resident macrophages in non-muscle tissue, highlighting the fact that macrophages have the ability to adapt to the local tissue environment and acquire tissue-specific functional identities.

### 3.2. Different Macrophage Populations That Are Present in Skeletal Muscle and Their Functions

Single-cell RNA sequencing (scRNA-seq) is used to quantify gene expression in specific cell populations by sequencing the entire transcriptome at the single-cell level [27,28]. As one of the key steps in single-cell transcriptome data analysis, unsupervised clustering plays a crucial role in identifying putative cell types and subgroups and interpreting cellular heterogeneity [29,30]. Therefore, transcriptome analysis by scRNA-seq analysis can be used to identify the functional properties of subgroups of macrophages in skeletal muscle. A recent study performed an unsupervised classification by pooling scRNA-seq data from young and old mice to gain insight into the subpopulations of skeletal muscle macrophages [3]. Unsupervised clustering identified eleven distinct macrophage clusters in the skeletal muscle of male mice, and gene ontology enrichment analysis confirmed that these clusters have enriched genetic programs related to reparative, proinflammatory, phagocytic, proliferative, lipid homeostasis, and senescence/aging functions (Table 1) [3]. Among them, the two largest clusters, cluster 0 and cluster 1, accounted for about half of the total macrophages, and both clusters showed high expression of M2-type genes and were closely related to reparative functions [3]. In contrast, M1-like macrophages were found to consist of eight distinct functional clusters (clusters 2–9) and exhibited the expression of mRNAs associated with various processes, including inflammation promotion (cluster 2 and 5), antigen processing and presentation through MHC class II molecules (cluster 2 and 7), cellular detoxification (cluster 3), phagocytosis (cluster 4 and 8), lipid homeostasis and cellular senescence (cluster 6), protein synthesis (cluster 7), and cell proliferation (cluster 9) [3]. These data suggest that M1-like macrophages exhibit greater heterogeneity compared to M2-like macrophages in mouse skeletal muscle.

In addition, the authors performed a supervised classification using the membrane markers LYVE1 and MHCII to complement unsupervised clustering and to obtain a more comprehensive view of the highly heterogeneous skeletal muscle macrophage group [3]. Skeletal muscle macrophages were classified into four subgroups, namely, LYVE1+/MHCII^hi^, LYVE1+/MHCII^lo^, LYVE1−/MHCII^hi^, LYVE1−/MHCII^lo^, based on the relative expression levels of LYVE1 and MHCII on their membrane [3]. LYVE1+/MHCII^lo^ macrophages overlapped significantly with clusters 0 and 1 from unsupervised clustering and displayed M2-like characteristics, with higher levels of mRNAs related to vasculature development and wound healing [3]. LYVE1−/MHCII^hi^ macrophages were the largest subgroup, accounting for 39.97% of the total macrophages, included clusters 2, 5, 6, 7, 9, and parts of cluster 3, and were overall more similar to M1-like macrophages for their involvement in antigen processing and presentation, cytokine production, and response to bacteria [3]. LYVE1+/MHCII^hi^ macrophages accounted for 36.65% of the total macrophages and were found to share mostly LYVE1+/MHCII^lo^ (M2-like) functions, but also LYVE1−/MHCII^hi^ (M1-like) functions [3]. LYVE1−/MHCII^lo^ macrophages contained clusters 4 and parts of clusters 3 and 8 and were associated with cytotoxic and phagocytic responses [3]. In particular, LYVE1+/MHCII^hi^ and LYVE1−/MHCII^lo^ macrophages have not been reported in skeletal muscle [25], and LYVE1−/MHCII^lo^ macrophages have not been reported in any other tissues [19,31]. About half of the LYVE1−/MHCII^lo^ subgroup exhibited robust phagocytic functions, and this subgroup, characterized as FcγRIV+/CD36+, consists of macrophages that were considered “super-phagocytic” in an early cancer study [32]. Therefore, further studies are needed to clearly confirm the function of this subgroup of macrophages in skeletal muscle.

In the same study, the authors further analyzed genes differentially expressed in young and old mouse skeletal muscle macrophages to identify aging-related changes in skeletal muscle macrophages [3]. In aged skeletal muscle, LYVE1− macrophages were more abundant than LYVE1+ macrophages, and consistent with this observation, the mRNA expression of *S100a8* and *S100a9*, encoding proinflammatory biomarkers, was markedly increased in macrophages of aged skeletal muscle [3]. S100a8 and S100a9 are known to act as biomarkers in various inflammatory diseases such as inflammatory arthritis and inflammatory bowel disease by forming heterodimers, and previous mouse model studies confirmed that blocking their activity reduced the inflammatory responses [33,34,35]. These results suggest that the expression levels of *S100a8* and *S100a9* in macrophages can be used as potential indicators of inflammatory conditions in skeletal muscle.

Another study utilized single-cell-based transcriptome analysis to identify the primary clusters of resident macrophages in the quadriceps and diaphragm [25]. The quadriceps and diaphragm contained the *CCR2* cluster [25]. The *CCR2* cluster showed abundant expression of *CCR2* and high expression of *MHCII* genes involved in antigen presentation, in both the quadriceps and the diaphragm [25]. When compared to the non-*CCR2* clusters, the *CCR2* cluster demonstrated a relatively low expression of “M2-like” genes [25]. Functional enrichment analysis revealed that genes differentially expressed by the *CCR2* cluster were enriched in antigen processing and presentation pathways in both the quadriceps and the diaphragm, while genes differentially expressed by non-*CCR2* clusters were enriched for pathways associated with phagocytosis and metabolism [25]. Additionally, based on the expression levels of the *MHCII* gene and the *LYVE1* gene, the *CCR2* cluster correlated with the MHCII^hi^Lyve1^lo^ skeletal muscle-resident macrophage subset, whereas the non-*CCR2* cluster correlated with the MHCII^lo^Lyve1^hi^ skeletal muscle-resident macrophage subset [25].

The quadriceps and diaphragm contain unique clusters in addition to the common clusters. The *CD209* cluster was found to be expressed only in macrophages of the quadriceps [25]. The *CD209* cluster exhibited high expression of a group of genes, including *MRC1*, *CD163*, *FCGRT*, *TIMD4*, *TSLP*, *FCNA*, and *FOLR2*, which are known to be upregulated in alternatively activated macrophages [25]. A functional enrichment analysis revealed that genes differentially expressed by the *CD209* cluster were enriched in the C-type lectin receptor activation pathways, which regulate immune responses [25]. C-type lectin receptors are a class of receptors responsible for recognizing and binding to various pathogenic molecules, including both exogenous pathogens and endogenous antigens [36,37,38]. To maintain tissue homeostasis in a steady state, myeloid cells rely on these receptors to detect and clear damaged cells and tissues, thus facilitating tissue repair functions [36,37,39]. Hence, the macrophages in the *CD209* cluster could exhibit heightened activity in clearing tissue damage in the quadriceps. In contrast, the *KLF2* cluster was found exclusively in the diaphragm and showed a significantly higher expression of stress-responsive gene groups compared to other clusters [25]. The results of the functional enrichment analysis revealed that genes differentially expressed by the *KLF2* cluster were significantly enriched in stress response-related pathways [25]. In particular, the diaphragm-resident macrophages were found to express significantly higher levels of stress response genes when compared to the quadriceps-resident macrophages [25]. The diaphragm undergoes constant contraction and relaxation during breathing, which leads to higher mechanical stress compared to the quadriceps, which do not experience the same repetitive movement. Therefore, these results support the hypothesis that the *KLF2* cluster enriched in the expression of stress response genes is more likely to be found in the diaphragm rather than in the quadriceps.

### 3.3. Obesity-Associated Insulin Resistance and Skeletal Muscle Macrophages

Although the increase of immune cells in adipose tissue during obesity is well known, it is still unclear whether immune cells infiltrate skeletal muscle in the context of overnutrition and insulin resistance. Importantly, the expression of inflammatory markers such as tumor necrosis factor-alpha (*TNF-α*) and C–C motif chemokine ligand 2 (*CCL2*) was increased in the skeletal muscle of obese humans and high-fat diet (HFD)-fed mice, indicating clear signs of local inflammation [40,41]. An increase in such inflammatory signals can occur partially in immune cells and is supported by the presence of increased macrophages within skeletal muscle, as detected by immunohistochemistry staining for F4/80 or CD68 and by the expression of the *CD11c* gene [42,43,44,45]. In addition, the expression of transforming growth factor beta 1, an anti-inflammatory cytokine primarily expressed by leukocytes including macrophages, was negatively correlated with the hemoglobin A1c and fasting plasma glucose levels [45]. These results suggest macrophage infiltration in skeletal muscle can potentially influence the development and progression of metabolic disorders such as obesity and diabetes.

Another study tested whether the local infiltration of skeletal muscle beds by innate immune cells occurs early in mice following high-fat diet feeding [46]. Mice fed an HFD for 1 week were characterized by glucose intolerance and increased muscle gene expression of lymphocyte antigen 6, *F4/80*, *TNF*, *CCL2*, and C–C chemokine receptor type 2 [46]. The major immune cells of the muscles were CD11c^+^CD11b^+^F4/80^+^ macrophages, which, on average, represented over 50% of the leukocytes [46]. Interestingly, the immune cell populations and their percent representations observed in the quadriceps muscle differed significantly from those observed in epididymal white adipose tissue or in blood [46], suggesting that the presence of immune cells in muscle is not merely a reflection of the presence of adipocytes or blood within the muscle beds. Additionally, *Mgl2* (M2 macrophages) expression in muscle leukocytes of mice fed a high-fat diet increased along with *CD11c* (M1 macrophages) expression from 1 to 10 weeks, and the increase in CD11c^+^ cells in muscle was found to be mainly due to the increase in macrophages [46]. Notably, the depletion of CD11c^+^ cells in an obese mouse model significantly reduced the production of TNF-α, interleukin 6 (IL-6), and monocyte chemoattractant protein-1 (MCP-1) in skeletal muscles and restored insulin sensitivity [41]. Also, muscles from obese and glucose-intolerant humans showed elevated levels of CD68 and CD11c, which are associated with poor glucose disposal and adiposity [46]. Moreover, CCL2 knockout (KO) mice showed significantly reduced macrophage infiltration in white adipose tissue and skeletal muscle compared to HFD-fed mice and improved insulin signaling [46]. These findings suggest that targeting CD11c^+^ macrophages in skeletal muscle has the potential to alleviate inflammation, restore insulin sensitivity, and improve glucose disposal. Furthermore, the reduced macrophage infiltration observed in CCL2 KO mice highlights the role of CCL2 in promoting macrophage accumulation in adipose tissue and skeletal muscle, while its inhibition may contribute to improved insulin signaling.

## 4. The Mechanisms of Macrophage-Induced Insulin Resistance in Skeletal Muscle

**Figure 2:**
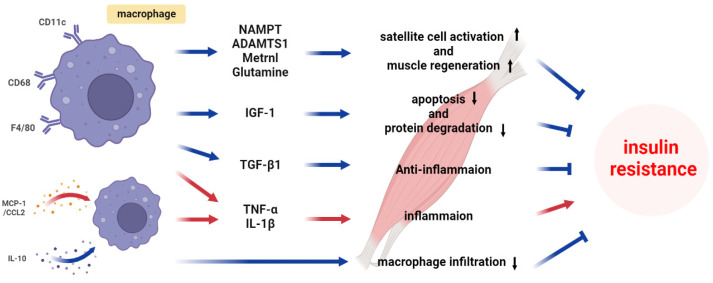
Mechanism of macrophage-induced insulin resistance in skeletal muscle.

Insulin resistance in skeletal muscle is a multifaceted phenomenon influenced by macrophages and the molecules they secrete [45,46,47,48]. Although the role of macrophages in the effects of insulin resistance on skeletal muscle is not well understood, several types of macrophages are distinguished, including M1 and M2 macrophages, and these different types of macrophages are closely related to insulin resistance and metabolic disease [3,8,46]. M1 macrophages produce pro-inflammatory cytokines, reactive oxygen species, and specific interferons, which can promote inflammatory responses and disrupt insulin signaling pathways, thereby contributing to the development or exacerbation of insulin resistance [8,49]. In contrast, M2 macrophages secrete anti-inflammatory cytokines and are involved in tissue remodeling, improved motility, functional angiogenesis, and the restoration of metabolic homeostasis [50]. Moreover, M2 macrophages can play a crucial role in reducing insulin resistance, restoring insulin signaling, and optimizing the overall metabolism [40,51]. Gaining a deeper understanding of the possibilities to modulate these distinct macrophage functions may be crucial in developing innovative therapeutic strategies for preventing and managing metabolic diseases associated with insulin resistance. This section aims to delve into the underlying mechanisms of insulin resistance by exploring the metabolic alterations induced by macrophages (Figure 2).

### 4.1. Classical Cytokines

Classical macrophage-secreted cytokines play a crucial role in the complex interplay between macrophages and skeletal muscle, influencing insulin metabolism [47,52]. The use of the Transwell co-culture method in a recent investigation provided valuable insights into the direct effects of activated macrophages on muscle insulin metabolism in cell models [53]. The findings demonstrated that lipopolysaccharide-activated RAW 264.7 macrophages significantly impaired insulin response in C2C12 myotubes, which was accompanied by elevated secretion of TNF-α and IL-6 [53]. Notably, TNF-α exhibited a dose-dependent effect in inducing insulin resistance, while IL-6 did not contribute to this response [53]. The underlying mechanism involved TNF-α triggering the activation of downstream proteins, such as ikappaB kinase and c-jun N-terminal kinase 1 (JNK1), ultimately resulting in the phosphorylation of IRS-1 at Ser residues [53,54,55]. Targeting TNF-α therapeutically holds immense potential to address insulin resistance and provide interventions to prevent inflammation-induced insulin resistance by suppressing TNF-α production. Furthermore, IL-6 has been implicated in insulin resistance through the activation of JNK 1/2, the accumulation of suppressor of cytokine signaling 3 mRNA, and increased protein tyrosine phosphatase 1B activity [56,57]. However, the lack of observed effects of IL-6 in this study indicates that the dosage and duration of IL-6 exposure can modify insulin sensitivity in skeletal muscle. Other studies have provided valuable insights into the crucial role of macrophages in facilitating muscle strength recovery during muscle atrophy, as evidenced by both in vivo and in vitro models [58]. Notably, the depletion of macrophages induced by etoposide in a rapid hindlimb unloading/reloading C57BL/6 mouse model was observed to impair muscle recovery [58]. Furthermore, in vitro, investigations utilizing a macrophage–myotube co-culture system demonstrated the efficacy of macrophage-released insulin-like growth factor 1 (IGF-1) in mitigating myotube atrophy and reducing myosin content [58]. Mechanistically, the activation of IGF-1 stimulates the anabolic mammalian target of rapamycin in the AKT pathway, inhibits glycogen synthase kinase 3 beta, inactivates fork-head box O, and suppresses apoptotic and degradative enzymes (muscle atrophy F-box and muscle-specific RING finger protein 1), resulting in enhanced protein synthesis and reduced protein degradation [59,60,61]. These intriguing findings also suggest the possibility of a potential link between IGF-1 and insulin resistance, highlighting the need for further investigation in future studies.

The modulation of macrophage infiltration into muscle tissue by cytokines plays a crucial role in influencing insulin resistance. In a study involving patients with T2D, the researchers observed the upregulation of the macrophage marker *CD68* and the cytokine *TNF-α* in skeletal muscle [49]. The expression of these markers showed a strong association with elevated plasma-free fatty acid levels and insulin resistance in patients [49]. To further investigate the impact of macrophage recruitment mediated by the chemokine MCP1 on insulin sensitivity, particularly in skeletal muscle, a mouse model with MCP1 overexpression under the control of the muscle creatine kinase (MCK) promoter was employed [49]. Within the gastrocnemius muscle of this mouse model, significant increases were observed in the expression of the macrophage markers *CD68* and *CD11c*, the pro-inflammatory cytokines *TNF-α* and *IL-1β*, as well as the chemokine regulated upon activation, normal T cell expressed and secreted (*RANTES*) [49]. These findings provided conclusive evidence of MCP1-mediated recruitment of macrophages. Additionally, the study revealed a notable impairment of insulin-stimulated AKT phosphorylation within the same muscle, suggesting that MCP1-mediated macrophage recruitment in skeletal muscle may play a crucial role in the development of T2D [49,60]. Another study conducted with MCK-IL10 mice, which are mice that specifically overexpress IL-10 in muscle, provided evidence that IL-10 acts as a positive regulator of muscle insulin sensitivity [51]. When exposed to a high-fat diet, the wild-type mice exhibited increased infiltration of macrophages in the muscle, as indicated by the presence of the macrophage-specific markers CD68 and F4/80 [51]. This infiltration led to the elevated expression of local cytokines, including *TNF-α* and *IL-6*, which can negatively impact muscle insulin signaling through inflammatory responses [51]. However, in MCK-IL10 mice, these effects were significantly attenuated [51]. Moreover, the ratio of phospho-JNK1 to JNK1, which was elevated in the wild-type mice fed a high-fat diet, was notably reduced in the MCK-IL10 mice, indicating protection against inflammation-induced insulin resistance [51,62]. Interestingly, in the MCK-IL10 mice, the increase in CCR2 expression, which plays a role in macrophage recruitment by binding to MCP-1 was completely prevented [51,63]. These findings suggest that IL-10 overexpression in muscle inhibits macrophage infiltration, indicating its potential to modulate muscle glucose.

### 4.2. Recent Progress in Understanding Macrophage-Secreted Factors

The objective of this section is to examine the role of newly discovered macrophage-secreted proteins and their influence on skeletal muscle function, with a specific focus on their implications for insulin resistance. In a recent study utilizing RNA-seq analysis, distinct subsets of macrophages localized within the injury site were successfully identified in a zebrafish muscle injury model [64]. Cluster 2 macrophages, one of the identified subsets, were found to secrete proliferative signals, such as the cytokine nicotinamide phosphoribosyltransferase (NAMPT) [64]. Notably, NAMPT is associated with the C–C motif chemokine receptor type 5, which is expressed in proliferating progenitor muscle stem cells [64,65]. These experimental findings demonstrated the potential of macrophage-derived niche signaling, particularly involving NAMPT, as a promising therapeutic approach to addressing skeletal muscle injuries and related diseases. Another study identified ADAM metallopeptidase with thrombospondin type 1 motif 1 (ADAMTS1) as an extracellular protein released at high levels from a specific subtype of macrophages called Ly6C^+^ macrophages, which are rapidly recruited to sites of muscle damage [66]. Co-culture experiments involving macrophages and primary satellite cells from Adamts1-overexpressing mice revealed a significant role for ADAMTS1 in initiating satellite cell activation, a critical process in muscle regeneration [66]. Moreover, the secretion of ADAMTS1 by macrophages was found to reduce the levels of the active region of the neurogenic locus notch homolog protein 1 intracellular domain in satellite cells, thereby inhibiting Notch signaling, a well-established regulator of satellite cell quiescence [66,67,68]. These findings provided insights into the regulatory mechanism of satellite cell activation by the novel protein ADAMTS1 and its implications for muscle regeneration. According to a recent study, the macrophage-secreted protein meteorin-like protein (Metrnl) is a crucial regulator of muscle regeneration and a promising therapeutic target for enhancing tissue repair [69]. The utilization of single-cell RNA sequencing allowed researchers to observe elevated levels of Metrnl in macrophage clusters within injured muscle tissue [69]. Furthermore, these researchers conducted experiments using macrophage-specific Metrnl KO mice, which demonstrated a noticeable reduction in muscle recovery [69]. These findings indicated that Metrnl plays a vital role in stimulating the expression of the *IL-10*, *IGF-1*, and *IL-6* genes within macrophages [69,70,71]. As a result, Metrnl promotes the differentiation of macrophages into an anti-inflammatory phenotype by activating Stat3, thereby inducing the production of IGF-1 [71,72]. This activation of IGF-1 directly impacts the proliferation of primary muscle satellite cells [73]. Collectively, the induction of an anti-inflammatory phenotype by the recently identified macrophage-secreted proteins, along with the activation of muscle satellite cells and the treatment of muscle damage, hold promise as a potential therapeutic strategy for conditions such as insulin resistance and T2D.

### 4.3. Metabolite

In a recent study exploring the impact of macrophage-derived metabolites on muscle health, compelling evidence has emerged regarding the crucial role of macrophage-derived glutamine in promoting satellite cell activation and muscle regeneration [74,75]. The study demonstrated that the absence of glutamine dehydrogenase 1 in macrophages led to a sustained elevation in the activity of glutamine synthetase, effectively preventing glutamine deficiency [74]. Moreover, when C2C12 cells were co-cultured with glutamine dehydrogenase 1-KO macrophages, the conditioned medium exhibited the ability to enhance the expression of the proliferation marker proliferating cell nuclear antigen and the differentiation marker myogenin [74]. Notably, this effect was accompanied by the activation of the mammalian target of the rapamycin pathway, which plays a critical role in satellite cell proliferation and differentiation [74,76]. These findings provide strong support for the significant influence of macrophage-derived glutamine on the differentiation potential of C2C12 myoblasts, suggesting that targeting glutamine dehydrogenase 1 could offer a promising therapeutic approach to promote muscle regeneration. Furthermore, the enhanced muscle regeneration process holds potential implications for improving insulin resistance and managing T2D, thereby opening new avenues for treatment.

## 5. Conclusions

Insulin resistance is a prominent feature of T2D, and skeletal muscle is an important site of insulin-mediated glucose uptake. The reviewed studies indicate that other subsets of cells within the skeletal muscle may have a significant impact on the development of insulin resistance. These subsets can have effects on various aspects such as tissue homeostasis, muscle growth, antigen processing and presentation, chemokine signaling modulation, and others. However, there are still many obstacles in this field. For example, the different subpopulations of macrophages and their functions within the skeletal muscle are not well understood. To overcome these problems, a deeper understanding of the various subpopulations and their functions is needed. Future research directions will include the identification of key molecular regulators that control macrophage polarization and function within skeletal muscle and evaluating the effects on insulin signaling pathways. Additionally, shedding light on the differences between muscle cells and other immune cells such as T cells and macrophages, is important for understanding the overall immune environment and metabolic health. Investigating the role of tissue microenvironments, including local cytokine environments, metabolism, and muscle fiber types, can help elucidate how macrophages in skeletal muscle contribute to insulin resistance. Additionally, implementing a precision medicine approach with in-depth, patient-specific analyses of macrophage populations, gene expression, and metabolic profiles is essential for developing personalized treatment strategies.

## Figures and Tables

**Table 1:** Macrophage populations in skeletal muscle and their functions.

Clusters	Key Functions	Supervised Classification Using Membrane Markers	Ref.
Clusters 0	Reparative	LYVE1+/MHCII^lo^	[3]
Clusters 1	Reparative	LYVE1+/MHCII^lo^	
Clusters 2	Inflammation promotion Antigen processing and presentation through MHC class II molecules	LYVE1−/MHCII^hi^	
Clusters 3	Cellular detoxification	LYVE1−/MHCII^hi^ LYVE1−/MHCII^lo^	
Clusters 4	Phagocytosis	LYVE1−/MHCII^lo^	
Clusters 5	Inflammation promotion	LYVE1−/MHCII^hi^	
Clusters 6	Lipid homeostasis and cellular senescence	LYVE1−/MHCII^hi^	
Clusters 7	Antigen processing and presentation through MHC class II molecules Protein synthesis	LYVE1−/MHCII^hi^	
Clusters 8	Phagocytosis	LYVE1−/MHCII^lo^	
Clusters 9	Proliferative	LYVE1−/MHCII^hi^	
Clusters 10	Reparative functions	-	

## Data Availability

The datasets generated for this study are available on request to the corresponding author.
